# Stress during first pregnancy increases seizure threshold in adult male offspring 

**Published:** 2014-01

**Authors:** Peyman Pajand, Mahmoud Elahdadi Salmani, Hooman Shajiee, Hasan Abiri, Iran Goudarzi, Kataneh Abrari

**Affiliations:** 1 Azad Islamic University-Damghan Branch, Damghan, Iran; 2 School of Biology, Damghan University, Damghan, Iran; 3 Institute of Biological Sciences, Damghan University, Damghan, Iran

**Keywords:** Kindling, Pentylenetetrazol, Seizure, Pregnancy, stress

## Abstract

***Objective(s):*** Stress induces many homeostatic aberrations which are followed by lifelong allostatic responses. Epilepsy is developed or influenced by different environmental factors, i.e. prenatal stress which makes many contradictory developmental changes in seizure threshold and intensity. We investigated the potential seizure response of the rat offspring to prenatal stress; the stress which was applied to their mothers.

***Materials and Methods:*** Nine day heterogeneous sequential stress (HSS) model was used before and during the first and before the second pregnancy. The kindling was induced using 13 IP injections of pentylenetetrazol (PTZ) every 48 hr to adult male Wistar rat's offspring.

***Results:*** The results of the present study demonstrated that, before pregnancy stress decreased the rate of kindling (*P*<0.05) in the offspring, while stress which was applied during pregnancy completely prevented kindling (*P* <0.001). Further, their convulsive latency was increased and tonic clonic seizure duration was decreased. In contrast, previous pregnancy and between pregnancies stress could not change kindling process. Although maternal separation stress did not change kindling development, it could increase convulsive intensities by elongating the duration of seizures (*P*<0.05) and reducing convulsion latency (*P* <0.05).

***Conclusion:*** It is concluded that stress detrimental effects could be prevented by stress which was applied around first pregnancy; however this beneficial effect is weakened by before second pregnancy stress.

## Introduction

Epilepsy is a common neurological disorder characterized by recurrent spontaneous seizures, with the approximate 0.5–0.7% of the population being affected worldwide (1). Our understanding of the epileptogenic process emanates largely from animal studies that have investigated status epilepticus (SE)-induced epileptogenesis (2). Among animal studies, kindling as a model of plasticity, gradually develops the susceptibility of the brain neuronal networks to epileptogenesis (3). Moreover, different kinds of stimulus from electrical to chemical can be used to evoke the kindling paradigm. The PTZ as a chemical convulsive agent is used to develop kindling in animals following repeated injections (4, 5). While each model uses different cellular components of neuronal networks in the brain, all result in a common mixture of increasing excitability, decreasing inhibition or both (6, 7).

Stress as a potential or actual threat imposes different changes in the animal behavior, which are achieved through the modulation of neuronal function involved in different aspects of hormonal and neural responses (8). Early age stress exposure affects the animal health more vigorously (9). Similarly, alterations during embryonic development in the uterine environment can produce permanent lifelong effects (9, 10). Furthermore, exposure to stress during pregnancy may be sufficient to induce permanent alterations in emotionality, cognition, neuroendocrine response and behavior (9), as well as development of hypothalamo-pituitary-adrenal (HPA) and other neuroendocrine axes. High levels of maternal corticosterone are developed due to stress (11, 12) which cause many abnormalities such as impaired feedback regulation of HPA axis (13, 14). Then, the vicious cycle will be completed due to decreased corticosterone receptors in hypothalamus and hippocampus (13). While the development of the fetus brain begins in the uterus, continues from birth to puberty and toward ageing, stress is likely to affect the brain development and modify seizure vulnerability in later life. Network circuitry, as one of the brain fine structures (15), emerges a determined level of excitability which is improved toward the end of the development. Many environmental factors can alter the susceptibility of these developed brain circuitry to epilepsy (16, 17), among these, the prenatal stress has deleterious effects on the development of this disease (18, 19).

Altogether, prenatal stress causes many behavioral and physiological dysfunctions such as stress vulnerability (20, 21). Although there are studies which highlight the effect of stress on seizure threshold and epileptogenesis (10), there is no consensus on the effect of prenatal stress on kindling development in offspring. Thus, the present study is designed to explore the effect of stress at different times around first pregnancy of mother rats and measure the kindling development in their mature pups.

## Materials and Methods

***Animals***

Virgin female Wistar rats (Iran Pasteur Institute, Tehran, Iran) weighing 180 g at the beginning of experiments were paired randomly with male rats. To ensure pregnancy, we checked the vaginal smears on a slide every day at 7:00 am. The day sperm were detected was considered as the gestational day (GD) 0 and delivery was expected to be 21 days later. After birth, male pups were selected (two male pups per mother to avoid litter effect), grew in their family cage normally until they were 2 months of age, when they were used for kindling procedure. Litters that were chosen for the second pregnancy were paired with male rats, one month after the first pregnancy. All experiments were done in accordance with the National Institute of Health Guide for the Care and Use of Laboratory Animals (NIH Publication No. 23-80, revised 1996) and modified based on the research ethics standards for the care and use of animals in Damghan branch-Islamic Azad University. Rats were housed as 5 animals per cage, except for pregnant dams which were kept alone in cage. All animals were kept under normal 12 hr light-12 hr dark cycle (lights on at 07:00 am) with free access to food and water. 

***Experimental groups***

Pregnant dams were randomly sorted into six experimental groups: (1) Before pregnancy stress (BPS), to which the stress was applied before the first pregnancy. (2) During the first pregnancy stress (DPS), (3) Between pregnancies stress (B-PS), to which the stress was applied before the second pregnancy. (4) Previous pregnancy stress (PPS), (5) Maternal separation stress (MS), as a standard and established postnatal stress model to be compared with other prenatal models, and (6) PTZ kindling non-stressed (because control group for the first and the second pregnancy and also maternal separation stress group were not significantly different, we pooled their data into PTZ non-stressed (PnS) group). 

***Stress protocol***

Two types of stress, including heterogeneous sequential stress (HSS) and maternal separation (MS) stress were used to compare seizure vulnerability in the offspring of stressed mothers and the stressed offspring, respectively.

HSS was applied during 9 days (22), in which several stress paradigms were included as follows: (1) Forced swimming for 10 min, (2) Restraint stress for 3 hr, (3) Water deprivation for 24 h, (4) Restraint at 4 ˚C for 1.5 hr, (5) Isolation from others for 24 hr, (6) Food deprivation for 24 hr, (7) Water deprivation for 24 hr, (8) Restraint at 4 ˚C for 2 hr, (9) Food deprivation for 24 hr (22). The HSS started 10 days before pregnancy in BPS and B-PS groups and 3 ± 1 days after pregnancy in DPS and PPS groups (about 4-14 days of pregnancy). 

MS is a model of stress to rat pups, started in the first 10 postnatal days. Mothers were picked up from the home cage and pups were left behind. Cages were kept in warmed water to hold the cage temperature at 37°C. Separation started from postnatal day 4 to 14 for 6 hr a day in the form of two 3 hr sessions which was interrupted for 10 min to let the mothers hugs the pups.

The HSS was used in different sorted groups of pregnant dams, while MS was only applied to pups of control, untreated dams. Neither the mothers nor the pups received stress in control non-stressed group and they were left undisturbed in the colony cage until kindling procedure.

***Kindling procedure***

Male Wistar offspring were selected to start kindling procedure, when they reached 150-170 g of weight (about two months old). PTZ (Sigma-Aldrich, USA), 40 mg/kg, was dissolved in 1 ml distilled water and administered intraperitoneally every 48 hr for 13 times. Each PTZ injected rat was monitored in a Plexiglas box (40*40*50 cm) for 20 min. Rats were considered kindled after 3 consecutive stage 4 or 5 of behavioral convulsions. Convulsions were scored (modified from the method of Racine *et al *(5, 23)) as follows: (0) no response, (1) ear and facial twitching, (2) convulsive waves through the body, (3) myoclonic jerks, (4) tonic-clonic (TC) convulsions and rearing, (5) generalized tonic-clonic seizures, turn on the side position and loss of postural control (5). Also, time average of thirteen convulsive stages in kindled and animals was used to compare excitability of the rat's brain (24).

**Figure 1:**
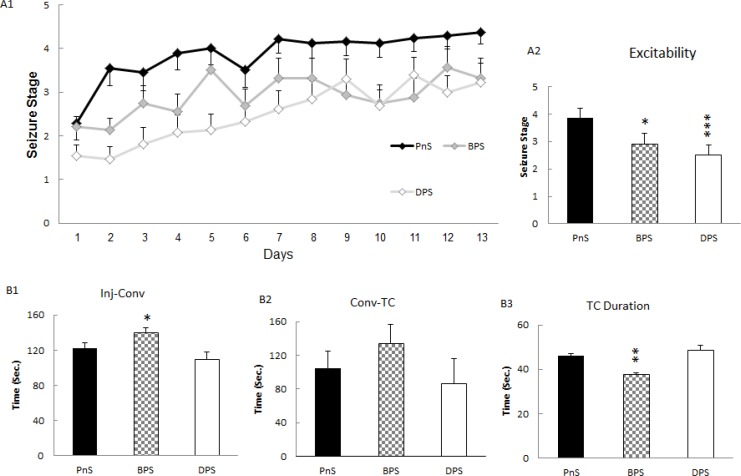
Before or during the first pregnancy stress prevented kindling development. Stress before the first pregnancy reduced (*P* <0.05) kindling rate and stress during it more profoundly decreased (*P* <0.05) kindling (A1). Total time average of PTZ kindling (inset) showed a decrease of excitability in stress before (*P*<0.05) and during (*P* <0.001) pregnancy (A2). Convulsion latency was increased in BPS (*P* <0.05), while it was without change in DPS (B1). Time from convulsion to TC seizures did not change significantly (B2). Duration of TC seizures decreased in BPS (*P* <0.01), but did not change in DPS (B3). * *P* <0.05; *** *P* <0.001, TC, Tonic clonic; PnS, PTZ non-Stressed; BPS, Before pregnancy stress; DPS, During pregnancy stress; PTZ, Pentylenetetrazol

Kindling score, 5, indicates the highest intensity of convulsions. So, increase in the intensity is not reflected in seizure stages. Therefore, we measured the number of TC seizures and three time periods starting from injection time, including: injection to convulsion (Inj-Conv.) or convulsion latency, convulsion to tonic clonic (Conv-TC) (25, 26) and tonic clonic duration (TC Dur.) (26, 27) for all convulsions. 

***Statistical analysis***

The average of latency to (Inj-Conv and Conv-TC) and duration of TC convulsions were calculated(28) for each group and were compared using one way ANOVA and LSD *post hoc *comparisons, when necessary. The time average of thirteen convulsive stages in kindled animals was used as an ordinal criterion to compare excitability of the rat's brain (24). Whole kindling period averaged ordinal values were compared using Kruskal Wallis which was followed by Dunn`s test, when appropriate. All of the statistical tests were performed by Statistica v.16 software. Data are presented as mean±S.E.M. and minimum significant level was set at *P*<0.05.

## Results

***Pregnancy has protective effects on the first pre-pregnancy stress***

After puberty and gaining the minimum weight of about 160 g, male adult offspring received repeated PTZ (40 mg/kg) injections. Comparing different stress effects using Kruskal Wallis test, showed a significant difference among kindling groups (H(4)=9.01, *P*=0.02). Then, Dunn`s method revealed that DPS elicited a prominent reduction in convulsive strength in adult offspring which was statistically significant (*P*<0.001, n=15). Similarly, BPS induced a less prominent but still statistically significant reduction of seizure intensity in kindling development (*P*<0.05, n=14). This data showed that applying stress around first pregnancy had a strong and significant increase in seizure threshold (Figure 1).

***Second pregnancy is incapable of reducing convulsions in the adult offspring***

**Figure 2:**
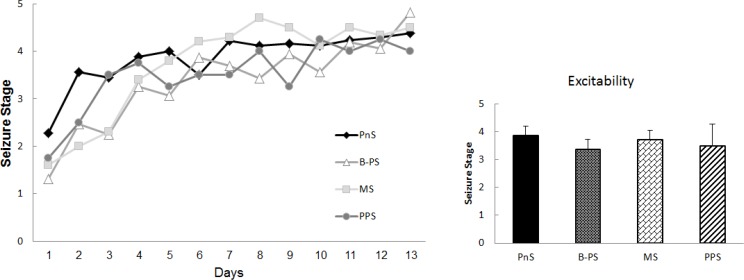
Kindling developmental rate increased due to later pregnancy stress. PPS increased kindling rate compared to before or during pregnancy stress and induced convulsive stages as PTZ kindling and maternal separation stress effect.

We compared the effect of stress between two pregnancies by applying the HSS before the second pregnancy. B-PS did not change kindling rate significantly (Dunn`s test, n=14, Figure 2), however, the average of the first five injections showed a reduction of kindling rate in its offspring (data not shown). In contrast, PPS (Dunn`s test, *P*<0.8, n=4) and MS (Dunn`s test, *P*<0.7, n=10), could not change the convulsive scores and hence the excitability, significantly. Comparing data from the first and the second pregnancy showed the disappearance of seizure reduction due to stress before the second pregnancy.

Inset; total time average of PTZ kindling in PPS and B-PS did not change significantly compared to PnS group. PnS, PTZ non-Stressed; PPS, Previous pregnancy stress; B-PS, Between pregnancy stress; MS, Maternal separation; PTZ, Pentylenetetrazol

***Seizure progression in the brain***

To have a complete look on the changes of seizure threshold, we compared latency and duration of seizures (25) as follows:

***Convulsion latency***

**Figure 3:**
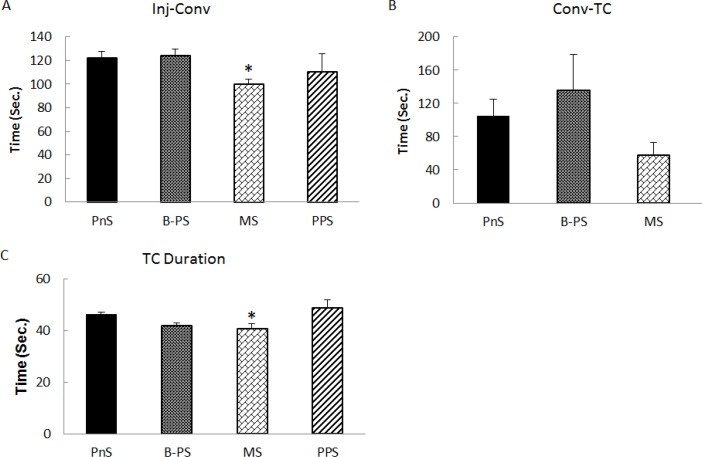
Maternal separation stress hastens TC seizures. Convulsion latency (*P*<0.05) (A) and convulsion to TC seizures (B) decreased in MS while PPS did not change compared to PTZ kindling. MS decreased TC seizure duration (*P*<0.05) while PPS did not change significantly.

The speed at which seizure spreads to motor cortex is a determinant of seizure severity. Comparing different stress treated groups using one way ANOVA showed a significant difference in convulsion latency (F_5,66_=2.76, *P*=0.02). LSD *post hoc* analysis revealed a significant increase in BPS (*P*<0.05, Figure 1-B1, n=16) but a reduction in MS (*P*<0.05, Figure 3A, n=7) compared to PnS group. Thus, BPS reduced the speed of seizure initiation in the brain while MS increased it.

***Convulsion to TC seizures***

Convulsion starts by brief jerks and continues to tonic clonic seizures. Analysis of variance showed no significant difference of convulsion progression among different stressed and non-stressed groups (F_5,58_=1.47, *P*=0.2, Figure 2).

***Tonic clonic seizure duration***

Severity of seizures is reflected in tonic clonic seizure duration and frequency. One way ANOVA analysis showed statistically significant difference between stress treated animals (F_5,58_=7.01, *P*=0.0005). LSD operation showed a reduction in BPS (*P*<0.01, Figure 1- B3, n=12) and also MS (*P*<0.05, Figure 3C, n=5) groups compared to PnS control animals. BPS group had short TC convulsions and most of the animals did not experience TC seizures. They did not develop kindling and so, mortality decreased prominently (data not shown). Unlike BPS, DPS did not change significantly (Figure 1-B3, n=14) and MS decreased TC duration unexpectedly (Figure 3C).

* P<0.05, TC, Tonic clonic; MS, Maternal separation; PnS, PTZ non-Stressed; PPS, Previous pregnancy stress; B-PS, Between pregnancy stress

## Discussion

The present study showed that stressed animals that their mothers were stressed before or during the first pregnancy are resistant to PTZ kindling development. Further, offspring of stressed mothers in the previous pregnancy or between pregnancies had a kindling rate similar to PnS group. Furthermore, we demonstrated that adult pups of stressed dams in previous pregnancy or between pregnancies exhibit longer and more frequent convulsions.

Stressed pregnant rats show many allostatic changes due to stress exposure which were represented in HPA axis as well as autonomic system sympathetic subdivision (29). We demonstrated that stress just before or during the first pregnancy protects the adult litter brain from PTZ induced kindling. This protection might be due to modulation of glucocorticoids in a way that neutralizes their maladaptive effects on the fetus brain. A probable mechanism consistent with this effect is stress induced conversion of corticosterone to deoxycorticosterone (DOC), 5 α-dihydrodeoxycorticosterone (5α-DHDOC) and allotetrahydrodeoxycorticosterone (THDOC), a GABA_A_ receptor-modulating neurosteroid with anticonvulsant properties due to acute prenatal stressors (30, 31). Therefore, metabolizing corticosteroids to ineffective substances will help the animal to develop allostasis and accordingly, to reduce epileptic excitability. In addition, we demonstrated an extra resistance against stress during pregnancy, which is not justified completely by the neutralizing theory. In a similar but less prominent way, prenatal stress starting from gestation days 5-12 did not change kindling development, while other stress models consisting of prenatal (10) or postnatal (32), increased kindling rate. As Brunton and Russel (2008) suggested, there is an enzymatic pathway in placenta which protects the fetus from detrimental stress effects. The placenta expresses 11β-hydroxysteroid dehydrogenase 2 (11βHSD2), an enzyme which converts corticosterone to inert 11-dehydrocorticosterone; thus lowers the concentration of corticosterone in fetus blood (33). Moreover, there is a persuading explanation for more pronounced reduction of seizure susceptibility in rat offspring due to stress during pregnancy, based on which, enhanced secretion of prolactin during pregnancy can reduce the activity of HPA (33) and therefore it could reduce stress induced effects. Thus, the effect of pregnancy is more than simply wiping out stress enhancing effects on epileptogenesis through glucocorticoids developmental trace (9). Altogether, the evidence shows a stress induced protection, rather than injury, with an enhancement during pregnancy which possibly improves the placental barrier and protects the fetus. 

Elevation of noradrenaline and corticosterone in blood affects the function and the structure of the mother's brain and passing the blood placental barrier, changes the fate of the fetus brain development (9). In the second pregnancy, maladaptive changes increase in mother's brain. Increasing levels of immune cytokines in the blood is likely to be a responsible mechanism (34). As previously shown, immune activation signiﬁcantly increased peripheral IL-1β and corticosterone levels and upregulated IL-1β mRNA expression within the cortex and the hippocampus (35). This immune response and also increased corticosterone level reduce the sensitivity of the hypothalamus to feedback glucocorticoids signals and consequently increase the HPA activity due to stress which in turn enhances the immune activation (36). We demonstrated that the protective properties induced by stress before or during the first pregnancy is no more effective for the offspring of the second pregnancy of the dams stressed in their first pregnancy. Additionally, we showed that stress even before the second pregnancy does not have protective effects. It is possible that cytokine production enhanced stress detrimental effects and as one consequence increased the passage rate of cytokines and other destructive agents through the placental barrier. Alternatively, this effect could be attributed to oxidative stress (37, 38) due to allostatic load, accumulated in successive pregnancies which could probably turn off the enzymatic activity of placenta. Another explanation could be found in a study by Edwards *et al* (2002) in which prenatal stress in mid/late gestation increased seizure vulnerability in the male offspring but disappeared through adulthood (10). This kind of seizure susceptibility could possibly justify our data showing an increased rate of kindling development in the offspring of mothers stressed between first and second pregnancies. The demonstration of late gestation stress induced increase of seizure predisposition is correlated to corticosterone increase near the end of pregnancy. Additionally, maternal HPA will become hyperactive due to successive pregnancies, from the first to the second pregnancy and will affect the fetal HPA development and hence offspring kindling development. Taken together, protective capability of pregnancy was reduced in the second pregnancy compared to the first one, whether the stress applied in the first or before the second gestation.

Like prenatal stress (39), postnatal events can give rise to high, chronic corticosteroid levels, but unlikely due to activation of the organism’s own HPA axis alone (40). Our data showed an increased rate of kindling and mortality in maternal separated rat pups. In accordance, Salzberg, *et al* showed a persistent vulnerability to epileptogenesis due to MS, but in females rather than males (32). This indicates sex differences in epileptogenesis, which needs further investigations in future studies. In another study, postnatal stress had no significant effect on afterdischarges and kindling rate in infant, but not adult offspring (10). Those studies are likely to justify the controversy of our data in TC duration in MS animals, while highlighting our finding of increased kindling rate which might be due to the increased corticosterone and HPA function in the stressed rat pups.

## Conclusion

Our study indicates that stress before the first pregnancy will be damped and the fetus is protected from stress. However, the next pregnancy of stressed mother or stress application to the next pregnancy will predispose offspring brain to developmental maladaptive changes and decrease potential defense mechanisms against stress.
